# A case of clinical stage I gastric cancer with a schwannoma on the left supraclavicular fossa suspected as Virchow’s node metastasis

**DOI:** 10.1186/s40792-022-01439-0

**Published:** 2022-05-13

**Authors:** Yoshihiro Hara, Kenichi Nakamura, Shiro Iwagami, Katsuhiro Ogawa, Hiroshi Sawayama, Masaaki Iwatsuki, Yoshifumi Baba, Yuji Miyamoto, Naoya Yoshida, Hideo Baba

**Affiliations:** Department of Gastroenterological Surgery, Graduate School of Medical Sciences, Kumamoto University, 1-1-1 Honjo, Kumamoto, 860-8556 Japan

**Keywords:** Schwannoma, Gastric cancer, Virchow metastasis, Gastrectomy

## Abstract

**Background:**

Gastric cancer is relatively prone to metastasis, although distant metastasis is rare during the early stage of disease. Here we report a rare case of schwannoma-associated Virchow metastasis of a patient with early-stage gastric cancer.

**Case presentation:**

A 73-year-old man, diagnosed with early-stage gastric cancer, underwent preoperative scrutiny, and was only suspected to have Virchow metastasis. Owing to atypical metastatic findings, a lymph node biopsy was performed to confirm the diagnosis and to determine the treatment strategy. The pathology results of the biopsy showed a diagnosis of schwannoma, the patient was judged to be surgically resectable, and a laparoscopic gastrectomy was performed to achieve a radical resection. The patient is currently under outpatient observation with no apparent recurrence.

**Conclusion:**

Systemic chemotherapy is generally administered according to the physician’s clinical judgment, although the results of a lymph node biopsy contribute to a more curative treatment. When nonspecific metastases are found, it is important to make a reliable diagnosis and to select a treatment that achieves a cure.

## Background

Gastric cancer is relatively prone to metastasis, and Virchow metastasis is one of the most common types of distant metastases that target the lymphatics. Other metastases are hematogenous liver metastasis, disseminated Schnitzler metastasis, and Krukenberg metastasis, which may be caused by multiple factors. Although such distant metastases are often found in advanced cancers according to their depth of invasion or presence in lymph nodes, certain cases of gastric cancer with distant metastases occur during the early stage of disease [1, 2]. Here we report a case of clinical stage-I gastric cancer with a schwannoma on the left supraclavicular fossa, which was suspected as Virchow’s node metastasis.

## Case presentation

**Fig. 1:**
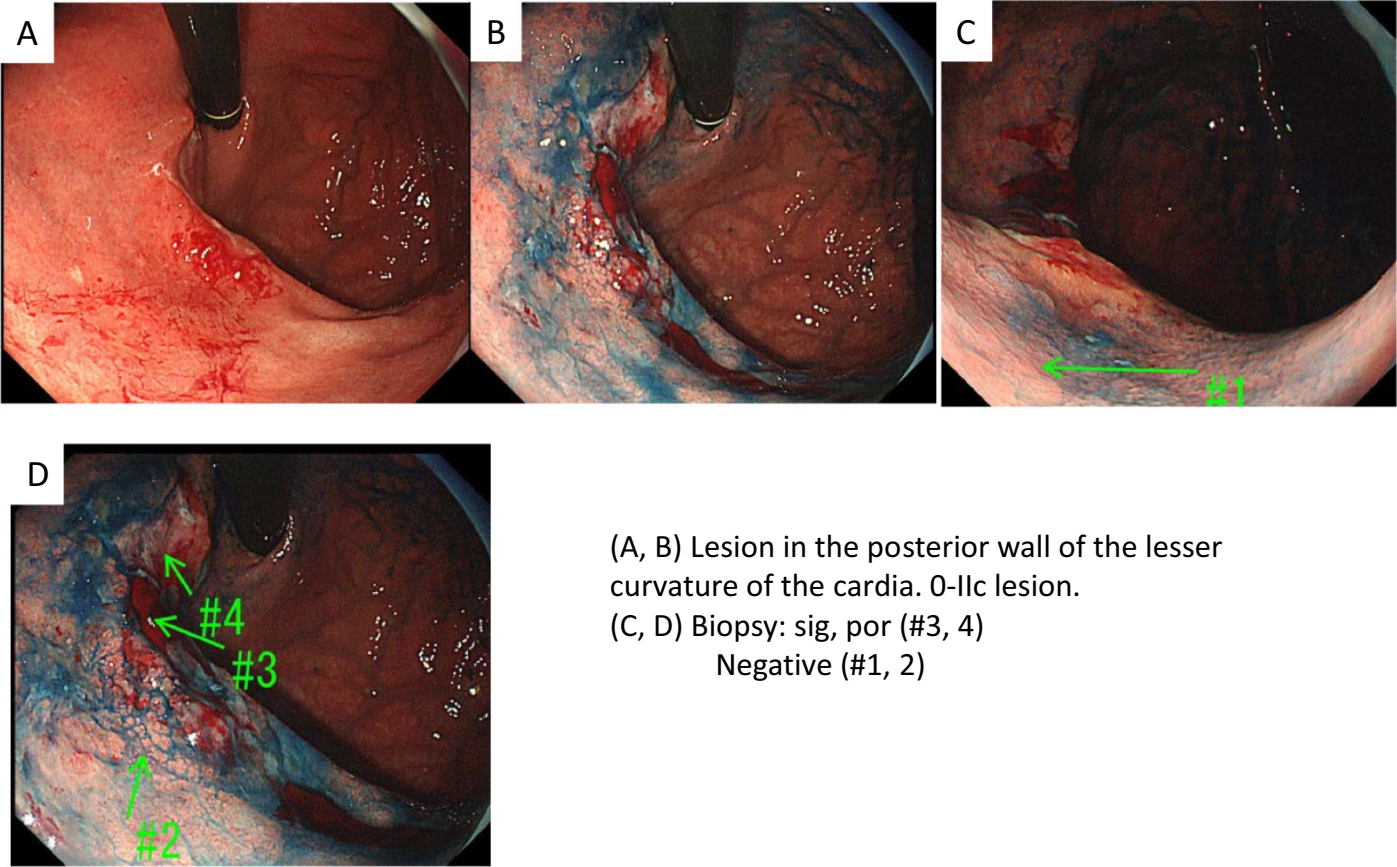
Upper gastrointestinal endoscopy

A 73-year-old man presented with a sore throat, hoarseness, and weight loss. Upper gastrointestinal endoscopy revealed a 30-mm type 0-IIc lesion on the posterior wall of the cardia of the stomach with an estimated depth of the muscularis propria (T2). (Fig. 1). An upper gastrointestinal series showed deformation of the posterior wall of the cardia, suggesting infiltration of the lesion into the muscularis propria (T2).

**Fig. 2:**
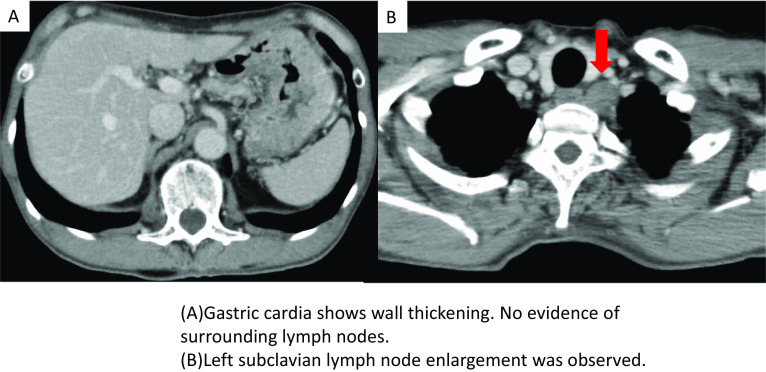
CT

**Fig. 3:**
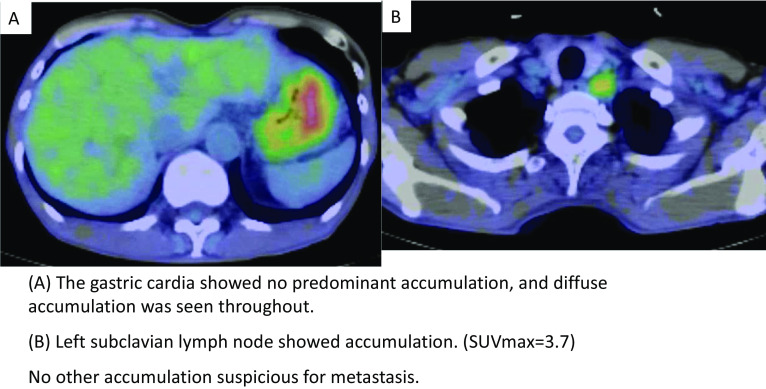
PET-CT

Biopsy showed a poorly differentiated adenocarcinoma with signet-ring cells. Contrast-enhanced computed tomography (CT) showed an enlarged lymph node in the left subclavian region, and a positron emission tomography-CT (PET-CT) scan showed mild FDG accumulation in the same lymph node (SUV-max_3.7_), which was suspected as a Virchow metastasis. (Figs. 2, 3).

There were no other obvious lymph node or distant metastasis findings. The cervical lymph nodes were not palpable, and blood tests detected normal levels of tumor markers and other variables (Table 1).

**Table 1:** Preoperative blood analysis

Variable (unit)	Value	Variable (unit)	Value	Variable (unit)	Value
WBC (× 10^3^/ml)	3.0	Alb (g/dl)	4.8	Na (mEq/l)	140
RBC (× 10^6^/ml)	4.30	BUN (mg/dl)	14.2	K (mEq/l)	4.1
Hb (g/dl)	13.9	Crea (mg/dl)	0.79	Cl (mEq/l)	103
Hct (%)	40.0	S-Glu (mg/dl)	99	Amy (U/l)	105
PLT(× 10^3^/µl)	192	T-Bil (mg/dl)	1.1	Ca (mg/dl)	9.3
Neut (%)	53.2	T-Bil (mg/dl)	0.1	CK (U/l)	84
Lymp (%)	38.5	AST (U/l)	26	CRP (mg/dl)	0.01
Mono (%)	6.3	ALT (U/l)	18		
PT (%)	120	LD (U/l)	241	AFP	3.8
APTT (%)	102	γ-GTP (U/l)	17	CEA	1.7
P-FDP (µg/ml)	4.5	ALP (U/l)	173	CA19-9	< 0.6
D-dimer (µg/ml)	1.7	CHE (U/l)	222	STN	38.0
TP (g/dl)	7.4				

*γ-GTP1* γ-glutamyltransferase, *Alb* albumin, *ALP* alkaline phosphatase, *ALT* alanine aminotransferase, *Amy* amylase, *APTT* activated partial thromboplastin time, *AST* aspartate aminotransferase, *BE* base excess, *BUN* blood urea nitrogen, *Ca* calcium, *CHE* cholinesterase, *CK* creatine kinase, *Cl* chloride, *Crea* creatinine, *CRP* C-reactive protein, *D-dimer* d-dimer, *Hb* hemoglobin, *Hct* hematocrit, *K* potassium, *LD* lactate dehydrogenase, *Lym* lymphocyte, *Mono* monocyte, *Na* sodium, *Neu* neutrophil, *P-FDP* plasma–fibrin–fibrinogen degradation product, *PT* prothrombin time, *S-Glu* serum glucose, *T-Bil* total bilirubin, *D-Bil* direct bilirubin, *TP* total protein, *WBC* white blood cell, *AFP* alpha-fetoprotein, *CEA* carcinoembryonic antigen, *CA19-9* carbohydrate antigen 19–9, *STN* sialyl Tn antigen

**Fig. 4:**
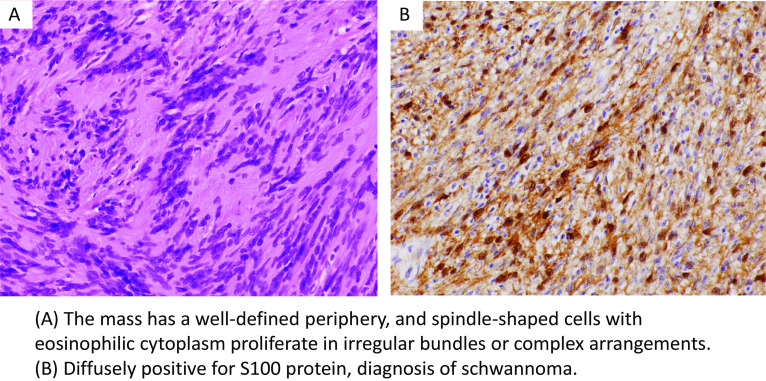
Histological findings of cervical tumor

We suspected Virchow metastasis of gastric cancer and therefore performed a left subclavian lymph node biopsy to determine the treatment strategy. Because of the close location of the cervical blood vessels and the risk of recurrent nerve injury, it was judged that a biopsy under local anesthesia would be difficult and was performed under general anesthesia. The tumor was an elastic, firm, well-defined limbed mass, approximately 20 mm major axis, located on the left side of the esophagus, dorsal to the left carotid artery and jugular vein. The tumor was completely removed. Pathological findings showed eosinophilic spindle-shaped cells that exhibited bundled and intricate arrangements. Immunostaining revealed diffuse expression of S100, which is diagnostic of a schwannoma (Fig. 4).

**Fig. 5:**
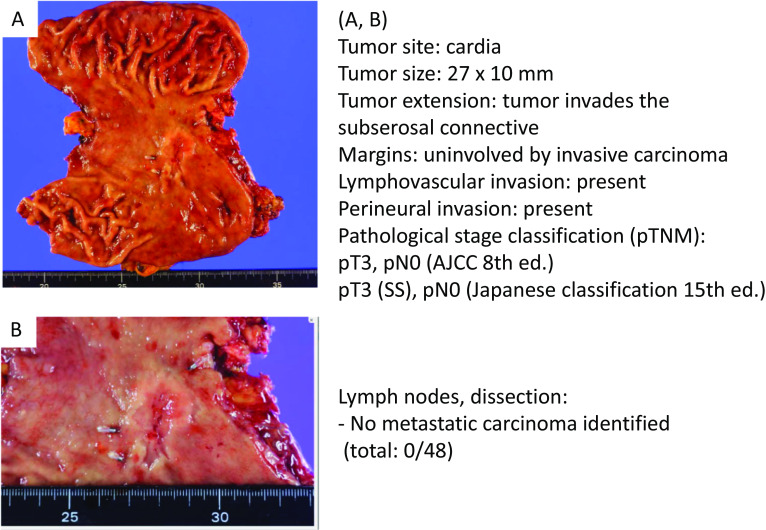
Gastric cancer

We, therefore, planned curative gastrectomy appropriate for the diagnosis of gastric cancer clinical stage I. One month after the biopsy, we performed laparoscopic proximal gastrectomy with D1 + dissection and double-tract reconstruction. Histopathological findings of the stomach were Type 2, por2 > tub2, pT3(SS), V1b, without detectable lymph node metastasis (pStage IIA) (Fig. 5).

The patient's postoperative course was good, and he began oral nutritional intake on the fourth postoperative day and was discharged on the tenth postoperative day. As of the completion of this case report (9 months after surgery), there has been no obvious recurrence, and the patient is being followed as an outpatient.

## Discussion

When early-stage gastric cancer is diagnosed, lymph node metastasis is not uncommon. According to the Gastric Cancer Treatment Guidelines 2018, Japanese Gastric Cancer Association [14], early-stage gastric cancer (SM1: < 500 µm from the muscularis mucosae) generates lymph node metastasis in ≤ 10% of cases, depending on the presence of an ulcer, cellular differentiation, and size. However, most metastases involve the perigastric lymph nodes of regional lymph nodes, and distant lymph node metastases are extremely rare. Among the 6 cases of Virchow metastasis associated with early gastric cancer reported in Japan, 4 patients survived for 3 to 7 months. Among the remaining 2 cases, 1 did not have detectable regional lymph node metastasis and survived for 5 years following resection and postoperative chemotherapy. The other patient underwent distal gastrectomy with extensive lymph node dissection of the para-aortic area and received postoperative chemotherapy, which contributed to the reduction of Virchow’s node metastasis. This latter patient survived 4 months after surgery without recurrence.

Although our present patient had advanced gastric cancer, we suspected only Virchow lymph node metastasis, and there were no other noncurative factors. We, therefore, considered an appropriate treatment strategy. The most frequent sites of overlapping cancers of the stomach, in decreasing order, are colorectal cancer, lung cancer, hepatocellular carcinoma, renal cell carcinoma, and lymphoma, although the association with schwannoma is not clear [3–8]. To the best our knowledge, the 3 published cases describe gastric cancer and sporadic schwannoma that developed in the retroperitoneum, para-aortic area, and liver [9, 11–13] (Table 2). Furthermore, Von Recklinghausen disease (neurofibromatosis), which is related to schwannoma, is frequently associated with malignancies, although rarely with gastric cancer [10].

**Table 2:** Patients’ characteristics

Case	Age	Gender	Pathological result of Gastric cancer	Region of tumor	Diagnosis of tumor	Reporter	Report year	Operation
	64	F	(Early stage)	Liver	Schwannoma	Wada Y, (12)	1998	Gastrectomy and partial hepatectomy
2	62	F	T1bN0M0,-, pStageI	Retroperitoneal	Schwannoma	Matsuhashi N, (9)	2013	Lap-distal gastrectomy and tumor resection
3	74	M	T1bN0M0, por, pStageI	Para-aortic lymph node	Schwannoma	Gakuhara A, (11)	2018	Lap-distal gastrectomy and tumor resection
4	73	M	T3N0M0, por, pStageIIA	Left subclavian lymph node	Schwannoma	Own case	2020	Lymph node biopsy Lap-proximal gastrectomy

*Lap* Laparoscopic

Schwannomas most often occur in the spinal cord, brain, limbs, neck, and rarely in the gastrointestinal tract [11]. Schwannomas mainly originate in the peripheral nerve sheath and are solitary. Generally, schwannomas are benign, although some may not be recognized as malignant tumors. Consequently, cases of recurrence and malignancies are reported, although they were actually benign, and therefore require follow-up. Gastrointestinal schwannoma, which is relatively rare, is considered a submucosal tumor that is easily treatable. Furthermore, a small number of cases of systemic diseases, such as sarcoidosis coexisting with early gastric cancer are considered difficult to distinguish from distant metastases, as in the present case [15].

Systemic chemotherapy is considered the standard treatment for distant metastasis and advanced lymph node metastasis of gastric cancer; and surgical resection, including preoperative chemotherapy, is considered an alternative. In such cases, when the primary tumor is clearly early-stage gastric cancer, other lymphadenopathies as well as metastasis must be considered. However, as in the present case, the pathological diagnosis is an advanced cancer, and diagnosis based on clinical and imaging findings alone is considered difficult.

Here we suspected only Virchow metastasis. We therefore administered nonspecific, diagnostic treatment and performed a cervical lymph node biopsy, leading to the diagnosis of a curable schwannoma. Instead, if the patient was diagnosed with stage-IV gastric cancer at the time of imaging, systemic chemotherapy may have been selected vs radical treatment. Therefore, it is important to consider other comorbidities and determine the diagnosis and treatment strategy if metastases or nonspecific masses are found that are inconsistent with the progression of the primary tumor.

## Conclusion

We treated a patient with a left subclavian schwannoma coexisting with gastric cancer, which made it difficult to preoperatively diagnose its progression.

## Data Availability

The datasets of this article are available on reasonable request.

## Abbreviations

CT: Computed tomography
PET-CT: Positron emission tomography-computed tomography
FDG: Fluorodeoxyglucose
SUV: Standardized uptake value
